# Evaluation of a pilot program that integrated prenatal screening into routine antenatal care in western rural China: an interrupted time-series study

**DOI:** 10.1016/j.lanwpc.2020.100075

**Published:** 2020-12-24

**Authors:** Xing Lin Feng, Chunmei Wen

**Affiliations:** a Department of Health Policy and Management, School of Public Health, Peking University, China; b Department head, Department of Maternal and Child Health, Health Commission of the Shaanxi Province, China

**Keywords:** Health service integration, Strategy of delivery, Supply- and demand-side policies, Public health vouchers, Antenatal care, Congenital abnormalities screening, Chinese public health program

## Abstract

**Background:**

We evaluated a large-scale pilot program in Shaanxi Province, western rural China, which integrated prenatal screening interventions for congenital abnormalities into routine antenatal programs.

**Methods:**

We surveyed 1,597 mothers who gave birth between 2009 and 2016. Adopting the interrupted time-series design, we evaluated the program's impact on awareness, coverage, and cost, by comparing two counties with only supply-side policies, and two counties from the neighbouring province with no policies; and among the two counties with both supply- and demand-side policies and the two counties with only supply-side policies. We adjusted the sampling procedure and women's background characteristics. We conducted subgroup analyses by women's education.

**Findings:**

After one year of implementation, the coverage of prenatal foetal aneuploidies and B-ultrasound screening rose by 25.0% and 23.5%. The program's supply-side policies attributed to 17.2 percentage points (90%CI 7.8–26.6%) and 27.3 percentage points (90%CI 16.2–38.5%) in coverage, and contributed to a higher median cost of 796.5RMB (90%CI 595.5–997.5). These significantly affected women with secondary education and above. However, the program's demand-side measures, that is, vouchers, seemed to be effective only in the mountainous regions, which raised awareness, and increased coverage of prenatal foetal aneuploidies screening by 28.6 percentage points (90%CI 13.4–43.8%), while not increasing costs. These significantly affected women with primary education and below. Education-related inequalities widened post-program in counties with only supply-side policies, but no inequalities existed in counties with demand-side policies.

**Interpretation:**

Shaanxi's program made a pilot study to other provinces of China to integrate antenatal services. Government subsidies might be more effective in targeting specific geographic locations and people with primary education and lower.

**Research in Context**

Evidence before this study

The global health community is calling for integrated maternal, new-born, and child health care. However, two latest systematic reviews from low- and middle-income countries (LMICs) concluded that there was still a striking lack of evidence on integration and delivery strategies. Prenatal screening of congenital abnormalities, that is, serum screening for foetal aneuploidies and B-ultrasound screening for structural malformation, is an important prophylaxis intervention. Our systematic search of the literature showed that no study has investigated the coverage and inequality of prenatal screening in rural China, particularly for the western part that encountered great challenges in promoting maternal, neonatal, and child health. Vouchers have been widely considered by policymakers and program managers in many LMICs to promote equity for antenatal care. However, systematic reviews revealed important literature gaps for implementation. First, no study has explored whether and how vouchers can help to facilitate the integration of other interventions into routine care. Second, no study has explored the mechanisms of how vouchers work, considering contextual factors.

Added value of this study

This study examined the first pilot program from rural China that attempted to integrate congenital abnormalities’ screening interventions into routine antenatal care. We found that after one year of implementation of this program, the coverage of the extended interventions increased substantially, which was independent of women's geographic access to health care, demographic factors, and socioeconomic status. On the one hand, the program's supply-side efforts increased the coverage of the extended interventions and contributed to a rising median cost. These significantly affected women with secondary education and above. On the other hand, the program's demand-side measures, that is, vouchers, seemed to be effective only in the mountainous regions and increased coverage of the extended interventions, while not increasing costs. These significantly affected women with primary education and below. In addition, education-related inequalities widened after one year's implementation in counties with only supply-side policies. However, in counties with demand-side policies, no inequalities existed.

Implications of all the available evidence

Other regions in China and Western Pacific may learn from this case to promote integrated antenatal care that built constructive accountabilities that link families, communities, and health providers. The Chinese government should also pay careful attention to the varied contexts while designing and tailoring voucher-based programs. It might be more effective to target specific geographic locations and people with primary education and lower.

Alt-text: Unlabelled box

## 1 Introduction

There has been a strong global commitment to universal health coverage, and the extension of essential preventive services is one of its central features [1]. Global consensus was required to provide integrated health services based on peoples’ needs [2]. However, previous studies focused mainly on the negative role of user fees that undermined access to health care [3]. Furthermore, related literature on how to provide services in a comprehensive and integrated manner in the context of frontline practices in low-resourced settings is scarce [4]. This applies particularly to maternal, neonatal, and child health (MNCH) [5], [6], [7]. Antenatal care serves as a platform to provide a continuum of interventions that could not only manage maternal risks but also improve child health outcomes [8]. Recent global public health guidelines recommend evidence-based interventions that should be integrated into routine antenatal care [9], [10]. These interventions include a series of prenatal screening services related to infectious diseases, malnutrition, and congenital abnormalities. However, the two latest systematic reviews on studies from low- and middle-income countries (LMICs) concluded that there was still a striking lack of evidence on the effect of integration and the delivery strategy to achieve universal coverage [11], [12].

Demand-side financing consists of a group of innovative tools to promote equity in essential health services [13], [14]. Vouchers have been widely considered by policymakers and program managers in many LMICs for antenatal care. As found in several systematic reviews, vouchers seem to be effective in improving coverage and targeting subsidies for disadvantaged groups [15], [16], [17]. However, two knowledge gaps exist [18], [19]. First, only a few studies have explored whether and how vouchers can make a difference in facilitating the integration of other interventions into routine care. Second, while vouchers, as a means of delivery, may reduce financial barriers to access and may acknowledge recipients of the services, few investigations have been conducted to understand the mechanism of vouchers, considering contextual factors.

**Fig. 1:**
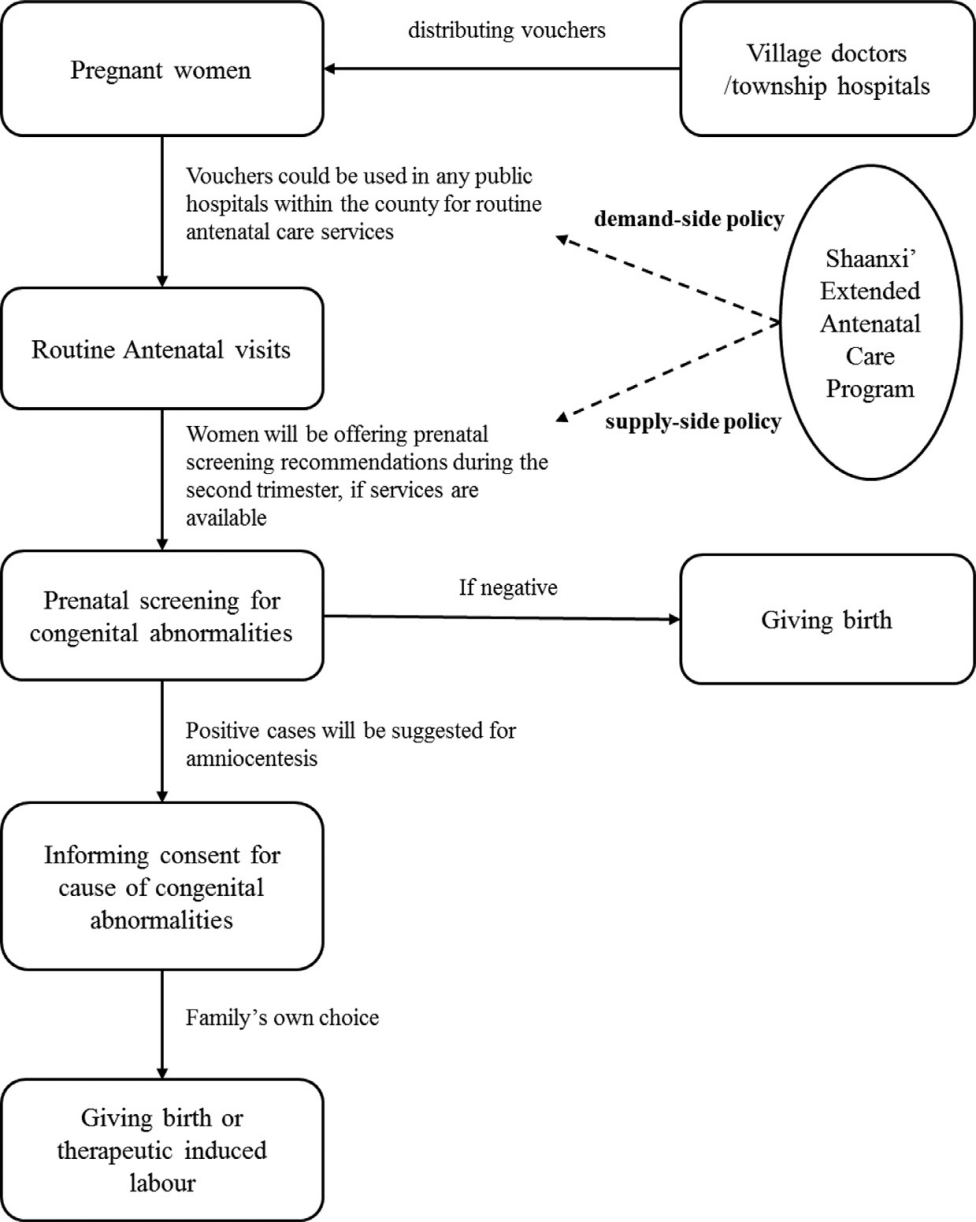
Patient pathway for antenatal care services in Shaanxi and Ningxia Province, Western China.

China has been scaling up its National Essential Public Health Program, which has been providing five free routine antenatal visits since 2009 [20]. The program builds on the WHO's recommendations to deliver a list of basic antenatal care services. In some western provinces such as Shaanxi and Ningxia, voucher-based systems were set up to deliver these services (Please see Appendix Table 1 for the procedure). Prenatal screening of congenital abnormalities, including screening of foetal aneuploidies and structural malformation, are important components of antenatal care. The Chinese government released guidelines for prenatal screening in 2003 (Please see Fig. 1 for patient flow). However, despite entry and pricing regulations, the government made little effort to scale up these services in rural areas. Surveys based in hospital settings show that the coverage was low even in the developed urban settings [21], [22]. Recently, the Chinese government has been calling for pilot reforms to promote integrated MNCH care [23]. The Extended Antenatal Care Program (“the Program” hereafter) in Shaanxi Province, Western China, was the first pilot in the rural areas. The Program, initiated on May 1^st^, 2015, aimed at integrating the two types of interventions that screen congenital abnormalities into routine antenatal care: prenatal foetal aneuploidies screening and prenatal B-ultrasound screening.

Specifically, Shaanxi Province implemented two types of managerial measures. Appendix Table 1 describes the policies in detail. Briefly, on the supply side, the government advocated universal uptake of prenatal screening services and established mechanisms to coordinate health providers and guide service procedures. Whereas on the demand side, the government directly subsidized families who used the extended interventions through the existing vouchers-based system within the National Essential Public Health Program. Notably, supply-side measures were universally introduced in Shaanxi, while demand-side measures (i.e., vouchers) for the new integrated services were piloted in only about one quarter of the counties predetermined by the provincial government, prioritizing poor counties and those having maternal mortality above the provincial average.

In this study, we evaluated the impact of the Program on coverage and equity. Comparing between counties, we tried to differentiate the impacts of the Program's supply- and demand-side managerial measures. By considering the contextual factors, we draw lessons for the Chinese government on how to proceed with integration and promote equity in similar settings.

## 2 Methods

### 2.1 Study design

According to data from China's national surveillance [24], the incidence of congenital abnormalities in China reached 15.7 per 1000 births in 2015, contributing to 10.8% of the nation's burden of neonatal mortality. The province of Shaanxi and Ningxia had similar infant mortality (around 5 per 1000 live births in 2015) and the incidence of congenital abnormalities compared to China's national average.

Using survey data, we conducted an observational study that compared between counties that had policies and similar counties that did not. First, to evaluate the impact of the Program's supply-side integration measures, we compared between two counties in Shaanxi Province where only supply-side measures were conducted, and two counties in Ningxia Province where integration had not yet commenced. We chose Ningxia because it is a neighbouring province of Shaanxi, and vouchers were used in both of the two provinces to deliver routine antenatal care. Second, to evaluate the impact of the Program's demand-side measures, we compared four counties within Shaanxi Province, where supply-side measures were universally carried out, but demand-side measures were only conducted in two of them, as a means to promote integration. In addition, the four counties within Shaanxi came out from two different contexts, where two neighbouring counties were from the mountainous regions and the other two neighbours were from the plain regions. For each pair, one had demand-side policies for integration and the other did not. (Please see Appendix Figure 1 for a map of locations).

### 2.2 Data sources

We surveyed mothers who gave birth during the 2009–2016 period in the six counties from July to August 2016 using a stratified, clustered sampling procedure. First, to define the stratum, we ranked all the townships in each county based on their travel time (distance) to the county hospital using Baidu Maps. We then classified the townships into three groups based on this travel time, where each group was a sampling stratum. Second, we sampled 3–4 townships within each stratum. The number of townships that were selected ranged from 10 to 12 for each county. For each township that was selected, we considered the distance of each village from the township maternal and child health doctor who was in charge of the National Essential Public Health Program. We then sampled at least two villages - one far from, and one near the township hospital. Third, we obtained lists of names of all the mothers living in the sampled villages from the local maternal and child health doctor. The lists of names contained the basic information - including name, address, date of birth, and telephone number - of all the mothers and children that were recorded in the annual report system. We investigated all the families on this list that were at home during the survey. Fourth, we defined seven age groups according to the mothers’ date of delivery before the survey: 0–6 months, 6 months to 1 year, 1 to 2 years, 2 to 3 years, 3 to 4 years, 4 to 5 years, and 6 to 7 years. The sample size was determined by the time window of the study. For each age group, we selected a minimum number of mothers for the study. For the 0–6 months’ age group, we surveyed at least 100 women, while for the other age groups, we surveyed at least 20 women in each group. We recruited and trained students from the author's institute, to interview the mothers using a structured questionnaire. Verbal informed consent was obtained from the participants of the study. The study was approved by Peking University (IRB00001052–14070).

In total, we approached 1615 mothers of which 16 (1.0%) refused to participate in the study, and the other 170 (10.5%) participants had important missing values. For the 170 participants, our student interviewers contacted them at least three times through phone calls to complement the missing questions, of which we excluded 18 questionnaires. We finally had 1597 (98.9%) valid questionnaires for this analysis. We input the data using the EpiData software version 3.0. Each questionnaire was double-entry and checked for inconsistencies. Data were then exported to Stata 13.1 for further recoding and analysis.

The first author designed the survey and developed the questionnaire with consultations to statisticians and epidemiologists in the author's institution. One section of the questionnaire requested the mothers to recollect the frequency, contents, and total costs of the antenatal care received. We built the following coverage indicators, defined as the proportions of women who declared having used such services: prenatal foetal aneuploidies screening, prenatal B-ultrasound screening, and at least five routine antenatal visits. We ascertained 2 cost-related indicators, i.e. the total medical and non-medical costs associated with antenatal care. Medical costs referred to the out-of-pocket payments to health providers to receive antenatal care, while non-medical costs referred to the costs of travel and accommodation that were directly related to the activities in seeking antenatal care.

In addition, the questionnaire investigated the respondents’ awareness of the government program and their benefit from it. These data comprised four indicators in this analysis: 1) knowing about the five free routine antenatal care visits; 2) having used antenatal care vouchers; 3) knowing about the Extended Antenatal Care Program; 4) having used the extended interventions free of charge. We also collected information on the following covariates: mothers’ education, households’ income per capita, mothers' health insurance, distance, parity, and age.

### 2.3 Statistical analysis

We described women's characteristics by types of interventions and regions. We analysed trends in awareness and coverage, adjusting confounding factors using Poisson regression with sandwich robust standard errors. We reported 90% confidence intervals (CIs) that accommodate higher type I errors because this is a pilot study with relatively small sample sizes. We adopted the interrupted time series approach to model the Program's impact [25]. The interrupted time series estimation was fulfilled using a regression analysis that modelled the change in level and change in trend from before and after the program, and the program impact was estimated halfway through the post-program period. We used ordinary least squares to model awareness and coverage; we used quantile regression to model costs to estimate the median. We adjusted the following covariates: mothers’ educational achievement, households’ income per capita tertiles, mothers' insurance coverage at birth, travel time to the county centre, mothers’ parity at birth, whether the women delivered in their home county, and mothers’ age at birth. We tested the sensitivity of the modeling strategy using the Difference-in-Differences approach with similar findings (Appendix Table 4–7). We conducted subgroup analyses stratified by the women's education and used the plot graph to demonstrate inequity patterns. We adjusted all monetary values relative to 2016 using consumer price indices. We used the “svy” command in STATA version 13.1, weighing each observation against the inverse of the sampling probability. Participants with missing values in any dependent variables and explanatory variables were excluded from the statistical analysis.

### 2.4 Role of the funding source

The funder of the study had no role in the study design, data collection, data analysis, data interpretation, or writing of the report. The corresponding author had full access to all the data in the study and all authors shared the responsibility for the decision to submit for publication.

## 3 Results

Table 1 and Appendix Table 2 summarize the background characteristics of 1597 mothers. A total of 715 mothers (44.8%) gave birth before May 1, 2015, which was when the Program was initiated, while the remaining 882 mothers (55.2%) gave birth after that. The median income per capita was 9329 RMB ($1376). In general, the mothers from Shaanxi were wealthier than those from Ningxia, while income did not vary much within Shaanxi and across regions. Health insurance (the New Cooperative Medical Scheme) coverage was 93.2% for the mothers from the six counties. In the mountainous counties of Shaanxi, 31.7% of mothers had to travel for more than one-and-a-half hours to reach the county centre, while in the plain counties of Shaanxi, only 51.6% of the mothers could reach the county centre within 30 min. Of the mothers, 93.6% achieved primary education and above, although the mothers in Shaanxi possessed higher educational levels, particularly those living in the plain areas. In Shaanxi, 2.5% of the mothers were adolescents, while the proportion was 16.9% in Ningxia; 97.2% of the mothers were delivering their first or second child in Shaanxi, while 28.5% of the mothers had delivered three times in Ningxia.

**Table 1:** Characteristics of the mothers that were investigated in six rural counties from Shaanxi and Ningxia Provinces, Western China, by type of policies, 2009–2016.

	**Counties from Shaanxi with both supply- and demand- side integration policy**		**Counties from Shaanxi with only supply-side integration policy**		**Counties from Ningxia without integration policy**	
	**N**	**%**	**N**	**%**	**N**	**%**
**Timing of birth**						
Before May 1st 2015	227	78.4%	234	79.6%	254	78.2%
After May 1st 2015	276	21.6%	286	20.4%	320	21.8%
Missing	0	0.0%	0	0.0%	0	0.0%
**Income quintile**						
Q1	118	23.9%	99	21.1%	314	51.8%
Q2	182	37.8%	185	36.7%	164	32.2%
Q3	202	38.3%	233	42.3%	96	16.1%
Missing	1	0.2%	3	0.6%	0	0.0%
**Travel time to county**						
<=30min	180	34.9%	141	25.5%	194	33.9%
30–60min	305	61.5%	224	44.0%	287	49.4%
>60min	18	3.6%	155	30.5%	93	16.7%
Missing	0	0.0%	0	0.0%	0	0.0%
**Mother's education**						
Illiterate	19	4.7%	14	3.0%	60	11.8%
Primary	327	67.2%	302	61.4%	458	80.5%
Secondary	94	18.6%	122	21.8%	36	5.3%
College and above	61	9.5%	79	13.8%	16	2.5%
Missing	2	0.4%	3	0.6%	4	0.7%
**Mother's age in giving birth**						
<20	13	3.0%	12	2.6%	97	16.8%
20-	321	64.9%	338	64.1%	373	68.6%
30-	169	32.1%	167	33.3%	99	14.6%
Missing	0	0.0%	3	0.6%	5	0.9%
**Parity in giving birth**						
1	219	42.9%	259	47.4%	171	29.4%
2	270	54.5%	244	50.1%	239	42.2%
3 and above	14	2.6%	15	2.5%	162	28.5%
Missing	0	0.0%	2	0.4%	2	0.4%
**Mother's ethnicity**						
Minority	2	0.4%	4	0.9%	482	84.3%
Han majority	501	99.6%	514	99.1%	92	15.7%
Missing	0	0.0%	2	0.4%	0	0.0%
**Place of delivery in home county**						
No	39	8.1%	55	11.0%	40	5.4%
Yes	462	91.9%	464	89.0%	533	94.6%
Missing	2	0.4%	1	0.2%	1	0.2%
**Legally married when giving birth**						
No	17	3.9%	21	3.2%	46	6.3%
Yes	486	96.1%	497	96.8%	528	93.7%
Missing	0	0.0%	2	0.4%	0	0.0%
**Mother's NCMS coverage when giving birth**						
No	23	4.5%	33	5.8%	46	7.9%
Yes	479	95.5%	483	94.2%	526	92.1%
Missing	1	0.2%	4	0.8%	2	0.4%
**County**						
Mei	244	48.3%	0	0.0%	0	0.0%
Qishan	0	0.0%	248	46.6%	0	0.0%
Baihe	259	51.7%	0	0.0%	0	0.0%
Xunyang	0	0.0%	272	53.4%	0	0.0%
Tongxin	0	0.0%	0	0.0%	271	47.6%
Yuanzhou	0	0.0%	0	0.0%	303	52.4%
Missing	0		0		0	

**Notes:**

1. A total of 1597 mothers that gave birth during the 2009–2016 period in six rural counties from Shaanxi and Ningxia provinces were investigated. The sampling design was adjusted using the “svy” command in STATA.

2. The households’ income per capita was grouped into three quantiles. The income was adjusted to its value in 2016, according to the annual consumer price indices in rural China. The cut-off points were 6669.3 RMB ($983.6) and 12,866.1 RMB ($1897.7) ($1 = 6.78 RMB in 2016), respectively.

3. NCMS, the New Cooperative Medical Scheme. This government-run health insurance system covers health care for families living in rural China.

**Fig. 2:**
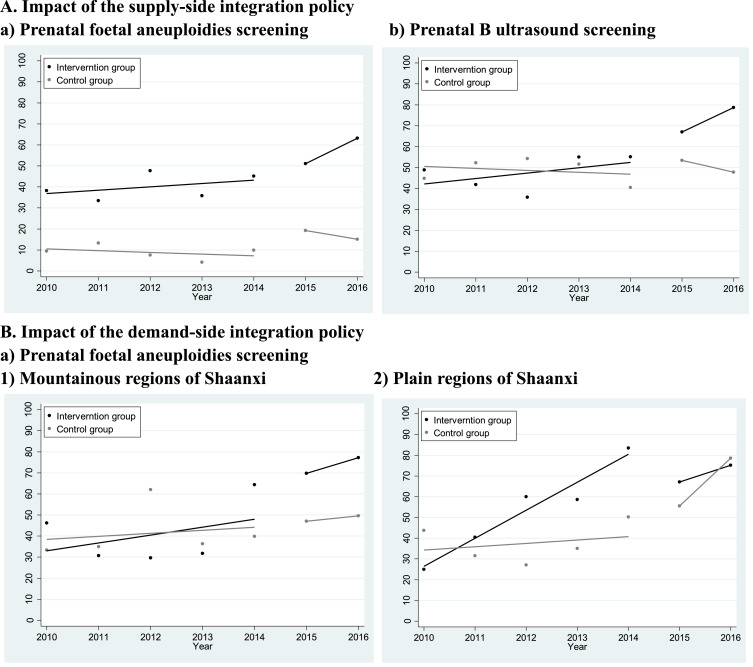

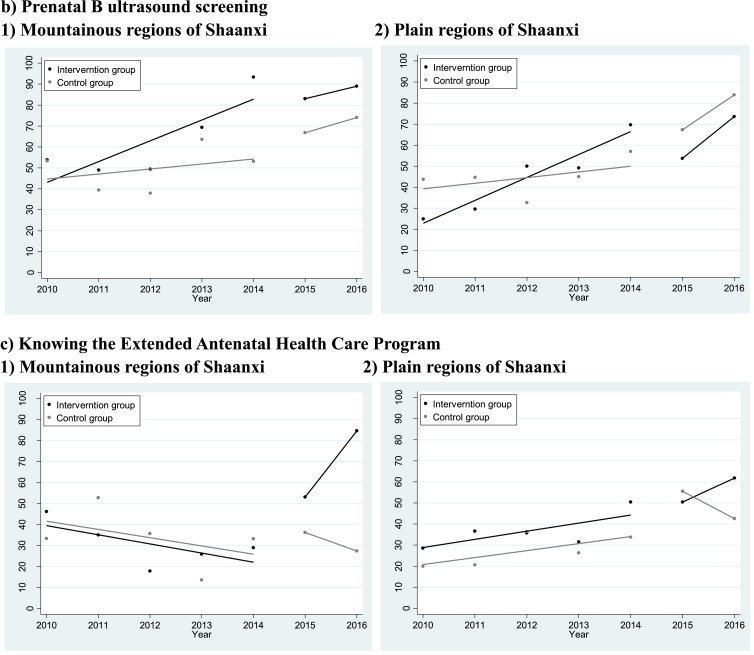
Pre- and post-program time series on coverage of the extended interventions and awareness of the Extended Antenatal Health Care Programs A. Impact of the supply-side integration policy a) Prenatal foetal aneuploidies screening b) Prenatal B ultrasound screening B. Impact of the demand-side integration policy a) Prenatal foetal aneuploidies screening 1) Mountainous regions of Shaanxi 2) Plain regions of Shaanxi b) Prenatal B ultrasound screening 1) Mountainous regions of Shaanxi 2) Plain regions of Shaanxi c) Knowing the Extended Antenatal Health Care Program 1) Mountainous regions of Shaanxi 2) Plain regions of Shaanxi Notes: 1. To model the impact of the supply-side integration policies, comparisons were made between two counties in Shaanxi Province where only supply-side measures were conducted, and two counties in Ningxia Province, where integration had not yet commenced. 2. To model the impact of the demand-side integration policies, comparisons were made between four counties within Shaanxi Province, where supply-side measures were universally carried out, but demand-side measures were only conducted in 2 of them, as a means to integration. In addition, the comparisons were made between neighbouring counties from 2 different contexts, i.e. the mountainous regions and the plain regions. .

Table 2 and Fig. 2 illustrate the pre- and post-program trends in awareness of the government program and coverage (Please see Appendix Figure 2 for the interrupted time series graphs for all outcomes and those by subgroups). In Shaanxi, the proportion of mothers who were aware of the Extended Antenatal Care Program rose from 33.0% to 52.7% after May 1^st^, 2015. Correspondingly, the coverage of prenatal foetal aneuploidies and B-ultrasound screening rose from 44.5% and 52.1% to 69.5% and 75.6%, respectively. In Ningxia, however, there were no obvious trends in coverage of these two types of interventions (see Table 3 for the control group). Table 2 also shows that the rising trends in coverage of the extended interventions in Shaanxi were independent of household income, travel time to the county, mother's educational achievement, insurance, age, parity, and place of delivery. After adjusting for these factors, the coverage of prenatal foetal aneuploidies and B-ultrasound screening increased by 52% (aRR=1.52 90% CI 1.38–1.67) and 44% (aRR=1.44 90% CI 1.33–1.56), respectively, after one year of implementation of the program. Awareness of and benefit from the program, and coverage of the two extended interventions did not vary across income, insurance, and travel time to the county, but were associated with mother's education (Appendix Table 3).

**Table 2:** Trends in awareness and benefit of the government antenatal care programs, and coverage of antenatal care services among the mothers that were investigated in the four rural counties from Shaanxi Province, Western China, 2009–2016.

	**Pre-program**	**Post-program**	**Crude RR**	**90%CI**	**Adjust RR**	**90%CI**
**Voucher-related performance**						
Knowing about the five free antenatal care visits	72.6%	81.7%	1.15	1.09,1.22	1.13	1.07,1.19
Having used antenatal care vouchers	77.3%	88.2%	1.13	1.08,1.19	1.11	1.06,1.16
Knowing about the Extended Antenatal Care Program	33.0%	52.7%	1.59	1.40,1.81	1.54	1.36,1.75
Having used the extended interventions free of charge	23.4%	25.8%	1.19	1.00,1.43	1.15	0.96,1.38
**Service-related performance**						
Prenatal foetal aneuploidies screening	44.5%	69.5%	1.58	1.43,1.74	1.52	1.38,1.67
Prenatal B ultrasound screening	52.1%	75.6%	1.48	1.36,1.60	1.44	1.33,1.56
At least five routine antenatal visits	81.7%	92.9%	1.12	1.08,1.17	1.11	1.07,1.15

1. 1023 mothers from four rural counties of Shaanxi province was included. The sampling design was adjusted using the “svy” command in STATA.

2. Since May 1^st^, 2015, Shaanxi province has been putting forward the Extended Antenatal Care Program.

3. Poisson regression was used in the adjust analysis. Sandwich robust standard errors were estimated to calculate 90% confidence intervals (CIs). The adjusted covariates include date of delivery, mothers’ educational achievement, households’ income per capita tertiles, mothers' NCMS coverage at birth, travel time to the county centre, mothers’ parity at birth, whether the women delivered in their home county, mothers’ age at birth, and county dummy variables.

**Table 3:** An interrupted time-series estimation of the impact of the supply-side policy of Shaanxi's Extended Antenatal Care Program on awareness, benefit, utilization, and costs of the antenatal care services, with Ningxia Province as the control group, by mothers’ educational achievement, using data from 4 rural counties in Shaanxi and Ningxia Provinces, Western China, 2009–2016.

	**Intervention group**		**Control group**		**Program impact**	
	Pre-program	Post-program	Pre-program	Post-program	Rate/RMB	90%CI
**Overall**						
**Service-related performance**						
At least five antenatal visits	77.8%	88.9%	65.4%	78.3%	−4.4	−13.3,4.6
Prenatal foetal aneuploidies screening	39.8%	64.1%	9.9%	16.1%	17.2	7.8,26.7
Prenatal B ultrasound screening	48.2%	75.2%	49.1%	51.1%	27.3	16.2,38.5
**Cost-related performance**						
ANC medical cost(median)	613.8	1416.8	156.3	200.0	796.5	595.5,997.5
ANC non-medical cost(median)	323.7	500.0	104.2	101.2	126.4	47.2,205.5
**Mother's education - primary or below**						
**Service-related performance**						
At least five antenatal visits	73.0%	83.7%	63.5%	80.3%	−2.4	−13.7,8.9
Prenatal foetal aneuploidies screening	39.9%	45.0%	8.8%	14.4%	2.3	−10.3,14.9
Prenatal B ultrasound screening	46.4%	66.8%	47.9%	49.4%	21.9	8.0,35.8
**Cost-related performance**						
ANC medical cost(median)	539.5	1012.0	156.3	200.0	577.8	393.2,762.3
ANC non-medical cost(median)	312.6	490.0	104.2	101.2	116.3	38.1,194.5
**Mother's education - secondary or above**						
**Service-related performance**						
At least five antenatal visits	89.4%	94.7%	89.1%	61.5%	10.6	−13.1,34.3
Prenatal foetal aneuploidies screening	41.2%	85.6%	25.0%	32.1%	35.0	5.0,64.9
Prenatal B ultrasound screening	52.3%	84.7%	67.1%	60.8%	42.7	9.3,76.1
**Cost-related performance**						
ANC medical cost(median)	818.4	2000.0	215.8	300.0	1105.3	158.6,2052.0
ANC non-medical cost(median)	333.4	506.0	111.2	121.4	46.5	−349.8,442.8

**Notes:**

1. 1094 mothers from four rural counties from Shaanxi and Ningxia Provinces was included. The sampling design was adjusted using the “svy” command in STATA.

2. In the Extended Antenatal Care Program, the government of Shaanxi adopted both supply- and demand-side policies. Since May 2015, supply-side measures have been universally conducted in Shaanxi, while demand-side measures were adopted in selected counties. In order to estimate the impact of the supply-side policies of the Program, we included two counties – Qishan and Xunyang - from Shaanxi where only supply-side measures were conducted in the treatment group and two counties from Ningxia, Shaanxi's neighbouring province, in the control group in this analysis.

3. The interrupted time series estimation was fulfilled using a regression analysis that modelled the change in level and change in trend from pre- to post- the program, and the program impact was estimated halfway through the post-program period. Additionally, we adjusted the following covariates: mothers’ educational achievement, households’ income per capita tertiles, mothers' NCMS coverage at birth, travel time to the county centre, mothers’ parity at birth, whether the women delivered in their home county, and mothers’ age at birth. We used ordinary least squares to model awareness and coverage; we used quantile regression to model costs in order to estimate the median.

4. *At least five antenatal visits, prenatal foetal aneuploidies screening*, and *prenatal B-ultrasound screening* were measurements of the actual coverage for these interventions. *ANC medical cost* was the median of the out-of-pocket payments that women declared were made to the health providers while using the antenatal care services. In addition, *ANC non-medical cost* was the median of the costs incurred by the women for their travel and accommodations while using the antenatal care services.

Table 3 reported the impact of the program's supply-side policies and that by women's educational achievement. It shows that these policies did not affect the coverage of five routine antenatal visits. However, these policies substantially improved coverage of the two types of extended interventions. As the model estimated, 17.2 percentage points (90% CI 7.8–26.7%) and 27.3 percentage points (90% CI 16.2–38.5%) for the coverage of prenatal foetal aneuploidies screening and B-ultrasound screening, respectively, could be attributed to the supply-side policies. The supply-side policies could independently explain a rise of 796.5 RMB ($117.5) (90% CI 595.5–997.5) in the median medical costs associated with antenatal care. In addition, the impact of the program's supply-side policies seemed to vary across the mothers’ educational achievement. For example, the model found that the policies had no impact in improving utilization of prenatal foetal aneuploidies screening amongst women with primary and below education (point estimates 2.3%, 90% CI 10.3–14.9%), but the impact on women with secondary and above education was 35.0% (90% CI 5.0–64.9%). The policies’ impact on prenatal B ultrasound screening seemed also larger amongst women with secondary and above education (42.7%, 90% CI 9.3–76.1%), than those with only primary and below education (21.9%, 90% CI 8.0–35.8%). The program's supply-side policies played a significant role in increasing the medical costs associated with antenatal care among women with secondary education and above as well.

Table 4 estimated the impact of the program's demand-side policies. The findings differed across the mountainous and plain regions. In the mountainous counties, the data showed that the program's demand-side policies increased the awareness and utilization of routine antenatal vouchers by 21.8 percentage points (90% CI 8.0–35.6%) and 13.4 percentage points (90% CI 0.6–26.2%), respectively. In particular, these policies contributed to a 45.8 percentage points rise (90% CI 30.2–61.5%) in the mothers’ awareness of the Program and an 18.2 percentage points rise (5.0–31.4%) in their benefit from it, respectively. Such policies also contributed to a 28.6 percentage points rise (90% CI 13.4–43.8%) in the coverage of prenatal foetal aneuploidies screening. Additionally, the model suggested that these policies had no role in increasing the median medical costs associated with antenatal care (90% CI −227.0 −743.8 RMB).

**Table 4:** An interrupted time series estimation of the impact of the demand-side policy of Shaanxi's Extended Antenatal Care Program on awareness, benefit, utilization, and costs of the antenatal care services, with a neighbouring county within Shaanxi as the control group, by regions, using data from 4 rural counties in Shaanxi Province, Western China, 2009–2016.

		**Intervention group**		**Control group**		**Program impact**	
		Pre-program	Post-program	Pre-program	Post-program	Rate/RMB	90%CI
**Mountainous region**	**Voucher-related performance**						
	Knowing about the five free antenatal care visits	44.7%	71.6%	78.9%	82.7%	21.8	8.0,35.6
	Having used antenatal care vouchers	58.6%	84.0%	77.9%	89.8%	13.4	0.6,26.2
	Knowing about the Extended Antenatal Care Program	28.8%	71.2%	34.2%	29.8%	45.8	30.2,61.5
	Having used the extended interventions free of charge	16.3%	23.2%	25.6%	15.2%	18.2	5.0,31.4
	**Service-related performance**						
	At least five antenatal visits	77.4%	96.4%	74.4%	81.0%	12.5	0.3,24.8
	Prenatal foetal aneuploidies screening	39.9%	80.0%	42.4%	52.4%	28.6	13.4,43.8
	Prenatal B ultrasound screening	64.7%	85.2%	48.8%	76.2%	−9.9	−23.8,4.1
	**Cost-related performance**						
	ANC medical cost(median)	1079.0	2000.0	607.2	1214.4	258.4	−227.0,743.8
	ANC non-medical cost(median)	312.6	303.6	431.6	566.7	−153.5	−318.6,11.7
**Plain region**	**Voucher-related performance**						
	Knowing about the five free antenatal care visits	90.0%	90.4%	76.4%	87.2%	−8.6	−20.2,2.9
	Having used antenatal care vouchers	93.5%	92.5%	79.1%	90.7%	−10.9	−20.5,−1.3
	Knowing about the Extended Antenatal Care Program	38.9%	56.2%	30.3%	52.9%	−4.5	−22.2,13.3
	Having used the extended interventions free of charge	31.6%	33.8%	20.7%	31.9%	−8.3	−24.0,7.3
	**Service-related performance**						
	At least five antenatal visits	94.5%	97.2%	82.5%	99.6%	−13.4	−21.0,−5.8
	Prenatal foetal aneuploidies screening	59.0%	68.1%	38.1%	76.1%	−27.0	−42.7,−11.2
	Prenatal B ultrasound screening	47.7%	64.4%	46.7%	80.5%	−16.7	−31.4,−1.9
	**Cost-related performance**						
	ANC medical cost(median)	1042.0	1012.0	625.2	1518.0	−755.9	−1186.7,−325.1
	ANC non-medical cost(median)	235.0	400.0	182.2	364.3	−50.7	−183.9,82.6

**Notes:**

1. 531 mothers from Baihe and Xunyang, two neighbouring counties from the mountainous areas of Shaanxi; 492 mothers from Mei and Qishan, two neighbouring counties from the plain areas of Shaanxi. In Baihe and Mei, both supply- and demand- side measures were conducted in the Extended Antenatal Care Program, while in Xunyang and Qishan, only supply-side measures were conducted. The sampling design was adjusted using the “svy” command in STATA.

2. The analytical approach is the same as that of Table 3 in terms of model specification, regression methods, and adjusted covariates.

3. The definition of dependent variables is the same as that in Table 4. Additionally, *knowing about the Extended Antenatal Care Program* measured the proportion of mothers who were aware of the Program. *Having used the extended interventions free of charge* measured the proportion of mothers who declared benefits.

In the plain areas of Shaanxi, however, the model suggested that the demand-side policies had no impact on the women's awareness of the program and their benefit from it. Surprisingly, the utilization of routine antenatal vouchers (−10.9 percentage points, 90% CI −20.5- −1.3%), coverage of five routine antenatal visits (−13.4 percentage points, 90% CI −21.0- −5.8), prenatal foetal aneuploidies screening (−27.0 percentage points, 90% CI −42.7- −11.2%), and prenatal B-ultrasound screening (−16.7 percentage points, 90% CI −31.4- −1.9%) rose faster in the control group rather than in the intervention group. Fig. 2 shows that the coverage of the two types of extended interventions rose faster in the intervention group before the program. In addition, the model suggested that the program's demand-side policies alleviated the costs associated with antenatal care by 755.9 RMB ($111.5, 90% CI −1186.7- −325.1RMB). However, no such effects were observed on non-medical costs (point estimate −50.7 RMB, 90% CI −183.9 - 82. 6 RMB).

Table 5 presents the estimation of the impact of the Program's demand-side policies related to women's education. In mountainous Shaanxi, these policies contributed to an increase of 24.5 percentage points (90% CI 10.3–38.7%) for women with primary education and below with regard to their declared benefit from the program, while such effects were statistically insignificant for women with secondary education and above (90% CI −37.8 – 20.5%). In terms of coverage, the Program's demand-side measures positively affected (40.3 percentage points, 90% CI 22. 5–58.2%) the coverage of prenatal foetal aneuploidies screening for women with primary education and below. However, no such effects were observed for women with secondary education and above (90% CI −37.0 – 25.3%). In plain regions of Shaanxi, the policy's differentiated effects across women's educational achievement were only observed in the coverage of prenatal foetal aneuploidies screening. The usage of such services increased more among women with secondary education and above in the control group rather than among those in the intervention group, whereas these effects were statistically insignificant among women with primary education and below.

**Table 5:** An interrupted time series estimation of the impact of Shaanxi's Extended Antenatal Care Program demand-side policy on awareness, benefit, utilization, and costs of the antenatal care services, by regions and by mother's educational achievement, using data from 4 rural counties in Shaanxi Province, Western China, 2009–2016.

	**Mountainous region**				**Plain region**			
	**Mother's education primary or below (*N*** **=** **390)**		**Mother's education - secondary or above (*N*** **=** **134)**		**Mother's education - primary or below (*N*** **=** **269)**		**Mother's education - secondary or above (*N*** **=** **220)**	
	Rate Diff./Yuan	90%CI	Rate Diff./Yuan	90%CI	Rate Diff./Yuan	90%CI	Rate Diff./Yuan	90%CI
**Voucher-related performance**								
Knowing about the five free antenatal care visits	19.3	2.8,35.8	21.2	−3.8,46.2	−13.4	−32.0,5.2	−1.1	−15.0,12.8
Having used antenatal care vouchers	14.8	0.8,28.8	11.3	−18.2,40.9	−13.9	−28.5,0.6	−7.5	−19.0,4.1
Knowing about the Extended Antenatal Care Program	52.7	36.4,69.0	25.5	−7.2,58.2	−20.7	−40.9,−0.4	11.5	−13.8,36.8
Having used the extended interventions free of charge	24.5	10.3,38.7	−8.7	−37.8,20.5	−14.2	−35.9,7.5	−4.6	−30.6,21.5
**Service-related performance**								
At least five antenatal visits	9.4	−5.3,24.10	17.1	−4.6,38.8	−16.3	−27.5,−5.1	−11.2	−18.6,−3.8
Prenatal foetal aneuploidies screening	40.3	22.5,58.2	−5.9	−37.0,25.4	−18.2	−44.6,8.2	−21.5	−42.4,−0.5
Prenatal B ultrasound screening	−3.7	−21.8,14.3	−16.3	−40.0,7.4	−9.4	−34.3,15.5	−10.1	−31.7,11.5
**Cost-related performance**								
ANC medical cost(median)	737.0	251.2,1222.9	−257.4	−1757.9,1243.0	−534.1	−1054.4,−13.8	−1035.0	−1874.5,−195.5
ANC non-medical cost(median)	−13.8	−207.6,180.1	−483.1	−978.8,12.5	−43.3	−232.2,145.5	−47.1	−298.4,204.2

**Notes:**

1. 1023 mothers from 4 counties of Shaanxi Province, Western China were included. The sampling design was adjusted using the “svy” command in STATA.

2. The analytical approach used and the definitions of the variables are the same as those in Table 3.

**Fig. 3:**
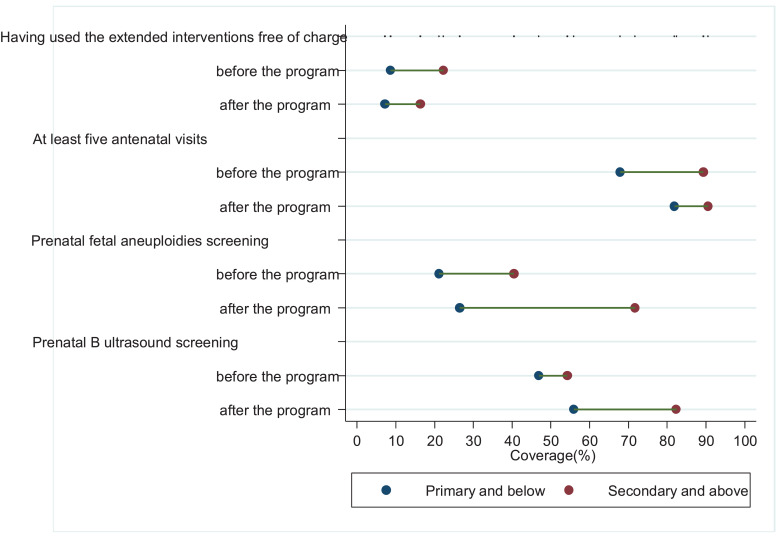
Trends in education-related inequity in benefiting from the government program and coverage of the interventions in the two counties where only supply-side policies were carried out, using data from Shaanxi province, Western China, 2009–2016. Notes: 1. In this analysis, 520 mothers from Xunyang and Qishan Counties, Shaanxi province were included. In the two counties, only supply-side policies were carried out in the implementation of the Extended Antenatal Care Program. 2. Poisson regression was adopted to test inequalities across the mothers’ educational achievement. Correspondingly, the P-values are 0.3106 and 0.7857 for *Having used the extended interventions free of charge* before and after the Extended Antenatal Care Program, respectively, 0.0013 and 0.0013 for *at least five antenatal visits* before and after the program, respectively, 0.5638 and <0.0001 for *prenatal foetal aneuploidies screening* before and after the program, respectively, and 0.4277 and 0.0002 for *prenatal B-ultrasound screening* before and after the program, respectively.

Fig. 3 shows that in the two counties where only supply-side policies were carried out, there was no difference across the mothers’ educational achievement with regard to their declared benefit from the government subsidies (*P* = 0.3106 and 0.7857 testing differences). In terms of coverage as well, there were no education-related inequities before the Program (*P* = 0.5638 and 0.4277 respectively). However, after the Program, women with secondary education and above had 45.2 (*P*<0.0001) and 26.5 (*P* = 0.0002) percentage points higher coverage of prenatal foetal aneuploidies screening and B-ultrasound screening, respectively, compared to those with primary education and below. In addition, persistent education-related inequalities were observed for at least five routine antenatal visits during the study period (P value=0.0013).

**Fig. 4:**
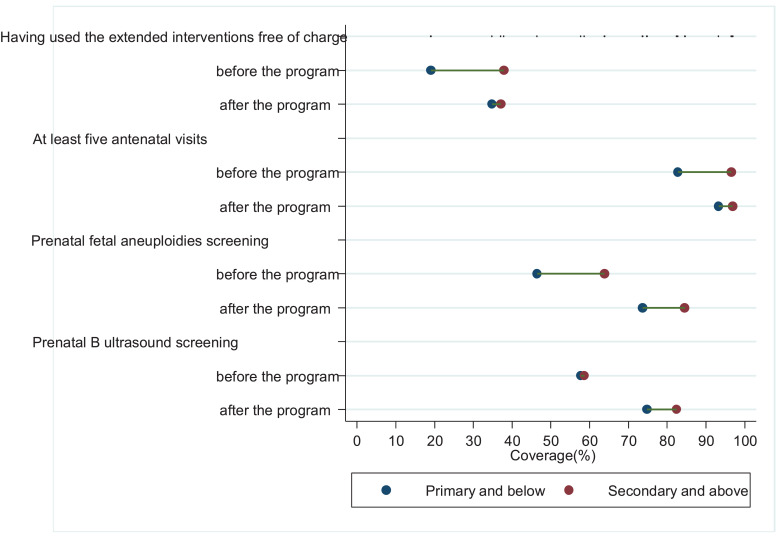
Trends in education-related inequity in benefiting from the government program and coverage of the interventions in the two counties where both supply- and demand-side policies were conducted, using data from Shaanxi province, Western China, 2009–2016 Notes: 1. In this analysis, 503 mothers from Baihe and Mei Counties, Shaanxi province, were included. In the two counties, both supply-side and demand-side polices were carried out in the implementation of the Extended Antenatal Care Program. 2. Poisson regression was adopted to test inequalities across the mothers’ educational achievement. Correspondingly, the P-values are 0.0030 and 0.7049 for *Having used the extended interventions free of charge* before and after the Extended Antenatal Care Program, respectively, 0.0004 and 0.1577 for *at least five antenatal visits* before and after the program, respectively, 0.0140 and 0.0268 for prenatal foetal aneuploidies screening before and after the program, respectively, and 0.9063 and 0.1233 for prenatal B-ultrasound screening before and after the program, respectively.

Fig. 4 illustrates different inequity patterns in the two counties – both supply and demand-side policies were carried out, education-related inequalities in the women's declared benefit from the government subsidies vanished after one year of implementation of the program (rate difference 18.9 percentage points, *P* = 0.0030, 2.3 percentage points, *P* = 0.7049 before and after the program, respectively). Similar patterns were observed in the coverage of routine antenatal care and the two types of extended interventions.

## 4 Discussion

The Sustainable Development Goals (SDGs) and the Global Strategy for Women's, Children's, and Adolescents’ Health are calling for countries to deliver integrated care for maternal, neonatal, and child health [26]. In this study, we illustrated a successful pilot program in western rural China that integrated prenatal screening interventions into routine antenatal care programs. The interrupted time series analyses found that the increase in coverage could be largely attributed to the Program's supply-side integration efforts. However, the Program's demand-side measures, that is, vouchers, seemed to be effective only in the mountainous regions, and affected women with primary education and below more significantly.

Prenatal screening of congenital abnormalities, including serum screening for foetal aneuploidies and B-ultrasound screening for structural malformation, are important secondary prophylaxis interventions. To date, we identified only two studies in urban China that analysed women's uptake of services [21], [22], and no studies investigating coverage and inequalities have been conducted in rural China. This issue is particularly significant for western rural China, which encountered enormous inequities and challenges in promoting maternal, neonatal, and child health [27]. In addition, emerging evidence has been reported from other LMICs that integrated HIV, syphilis, TB, and malaria screening interventions into routine antenatal services [11], [12], but little is known about prenatal screening. Using data from a large-scale, real-world pilot program, this study presented a new and unique case from western rural China, which attempted to integrate congenital abnormalities screening interventions. This is important, given the rising burden of congenital abnormalities, which affects child mortality and morbidity, in China and other LMICs [28]. The prevention and control of major congenital abnormalities has been prioritized by the Chinese government and society, and the screening of congenital abnormalities has been incorporated into China's national program since 2003. However, given the areas’ limited managerial and financial capacity, this program was vertically implemented in other provinces. To our knowledge, Shaanxi was the first province to integrate this vertical program into routine antenatal care in rural China. China's National Essential Public Health Program, which aims to universalize routine antenatal care, has existed for a decade, but coverage is not yet universal, and questions remain for policymakers on how to extend coverage. We found that coverage of routine antenatal care increased fast in Shaanxi during the post-program periods. There were no government programs that extend prenatal screening interventions in this province. This finding suggests that integrating more cost-effective interventions may help to improve routine services as well. With an annual per capita income of 8689 RMB ($1282), the province of Shaanxi has a population of 37.9 million, and 46% of them live in rural areas [29]. The lessons drawn in this setting may facilitate implementation in other rural settings of China.

Our survey shows that the coverage of both types of prenatal screening interventions had already been rising with a faster speed in the counties that were to receive demand-side interventions (vouchers) in plain regions of Shaanxi, which explains why we found an “inverse” impact of vouchers in this setting. Coverage of B-ultrasound screening rose fast in the mountainous regions of Shaanxi before the Program as well. Foetal aneuploidies screening services were newly introduced technologies in rural China, whilst the B-ultrasound scanning service (without standard clinical protocols for screening congenital abnormalities) was already available before the program. This helps to explain the differences across interventions. Hospitals may have incentives to import new technologies to attract more patients. However, whether the government chose counties with higher coverage to put forward subsidies is unknown. Further research is warranted to understand how technology infusion per se may affect coverage in rural settings.

While integration did play a role in improving coverage, our data showed that supply-side integration efforts alone might come at costs that undermine equity, consistent with experiences from other settings [3], [13]. International experiences illustrate the inverse care law, which states that the wealthier population benefits more from the new medical interventions[30]. This argument supports advocating demand-side subsidies for health care. With this stance, literature from the LMICs consistently reports that user fees hinder coverage and equity in essential public health services [3]^,^ [31]. Consequently, vouchers were typically considered as means to remove financial barriers [32]. However, in the specific context of our study, we found that households’ income and women's health insurance coverage were not associated with the uptake of the extended interventions, suggesting that families’ capacity to pay may not take the most important role that affects utilization.

Importantly, we found that women with higher educational achievement, that is, more knowledgeable women, were more likely to uptake the new interventions. The effects of vouchers varied across contexts and women's education as well, which were independent of women's wealth level, location, and other socio-demographic characteristics. These findings question the mechanism of how the voucher works in this specific context. In fact, in the plain regions, we found that vouchers had no effect on women's knowledge, that is, women from the county that had only supply-side policies had similar level and trends in awareness of the government program; whilst in the mountainous settings, where one-third of women had to travel more than an hour and a half to reach the county hospital, vouchers took a substantial effect in promoting knowledge of the government program. In the Program, the county maternal and child hospital took the responsibility to advertise the new interventions. Information was released in public media, such as TV, newspapers, and radios, or in the form of posters and booklets that were distributed in the location of county maternal and child hospital. Women living far might have found it harder to access these channels but the process of distributing vouchers make a chance for them to receive such information directly. This might explain why the impact of vouchers in the mountainous areas was greater among women with primary education and below, compared to those with secondary education and above, because the latter might be more likely informed by the public media. Taken together, vouchers’ effectiveness may stem from targeted acknowledging the recipients of the services rather than removing financial barriers. Global debates continue on whether and how to target while implementing demand-side measures, where means-test for the poor are costly and often ineffective in LMICs [18], [19]. Education certificates are more easily assessed in Chinese rural settings, which may offer a new approach for targeting. Our findings suggest that in contexts similar to Shaanxi, vouchers that target remote areas and the less educated, seem to be a more effective choice for policymakers and program managers. More experiments are warranted to better understand the mechanisms.

This study has several important limitations. First, this was an observational study that was based on a natural experiment from a government pilot program, we are not able to draw causal conclusions. We intentionally chose counties for the investigation. Although geographic factors were considered in the design and the comparisons were made between neighbours, these counties may still vary in background and baseline characteristics. To tease out the policies’ impact, we used an interrupted time-series approach that further adjusted demographic and socioeconomic factors, which might help to reduce biases [25]. Second, we used a stratified, clustered sampling procedure in the survey. Although we sampled nearly all the townships for county representativeness, we still have the risk of sampling errors. Moreover, we ascertained several dependent variables simultaneously, but the study design did not allow multiplicity. Third, because this is a pilot program and routine surveillance data were not available when the study was conducted, we were not able to evaluate the health outcomes of the program. Future work is warranted. Last but not least, survey data may have recalling biases.

## Conclusions

The other provinces in China could learn from Shaanxi's pilot program that intended to deliver integrated antenatal care to women living in rural settings. This program has made good progresses to build constructive accountabilities [33] that coordinate community-based institutions and county-level health providers, and effectively link families. The Government should pay careful attention to the varied contexts while designing and tailoring voucher-based programs. Rural Shaanxi's case suggests that it might be more effective to target specific geographic locations and people with primary education and lower.

## Authors contribution

Xing Lin Feng designed the research, organised the survey, analysed the data and wrote the paper. Other authors participated in the critical interpretation of the findings.

## Funding

The evaluation study is funded by Newton Advanced Fellowship jointly supported by the 10.13039/501100000691Academy of Medical Sciences and 10.13039/501100001809China National Natural Science Foundation (71761130083), and China National Natural Science Foundation Excellent Young Scientist Program (71422009). Both are fellowship projects that support the PI to do basic research in China. The funders played no role in this study.

## Data sharing statements

Individual participant data that underlie the results reported in this article will be available, with researchers who provide a methodologically sound proposal, beginning 9 months and ending 36 months following article publication. Please contact the corresponding author at fxl@bjmu.edu.cn.

## Declaration of competing interest

Xing Lin Feng declared no conflicts of interests.

Chunmei Wen works in the Health Commission of the Shaanxi Province. She is the department head in charge of maternal and child health of Shaanxi Province. She led her team in designing and implementing the Extended Antenatal Care Program in Shaanxi.

## Appendix Supplementary materials

Image, application 1

Image, application 2

Image, application 3
