# Supplementary material for: Evaluation of a pilot program that integrated prenatal screening into routine antenatal care in western rural China: an interrupted time-series study
Source: Lancet Reg Health West Pac. 2020 Dec 24;6:100075. doi: 10.1016/j.lanwpc.2020.100075 (PMC8315490; doi:10.1016/j.lanwpc.2020.100075)
Supplement: Supplementary file 2 [file mmc2.docx]

**Appendix Table 1 Service delivery of the National Essential Public Health Program for routine antenatal care in the rural areas of Shaanxi and Ningxia Provinces, Western China, 2009-2016**

| **Services** | **Designated providers** | **Delivery procedures** | **Household's payment to providers** | **Government's reimbursement to providers** |
| --- | --- | --- | --- | --- |
| 1. Collecting pregnancy information | Village doctors/township hospitals | Village doctors collected pregnancy information and reported it to the catchment township hospitals. The township hospitals reported the pregnancy information to the county health department and applied for vouchers. | Free | Budgetary payment to village doctors with adjustments based on annual performance ratings. The county project offices coordinated the annual performance rating. |
| 2. Establishing structured antenatal care record booklets and distributing vouchers to pregnant women | Township hospitals | The township hospitals distributed vouchers. | Free | Budgetary payment to providers based on the counts of vouchers collected by each provider. The county project offices coordinated this. |
| 3. Five routine antenatal consultations with specified routine examinations and tests | Any local county or township hospital that was certificated by the county project office | The families were free to choose among the designated providers for antenatal care. The families could use vouchers to cover the costs of the designated examinations and tests. | Free for the specified services that were included in the national program. Providers may charge families for extra services or tests that are not covered by the national program. |  |

**Policy settings**

1. **The National Essential Public Health Program and routine antenatal care**

In the National Essential Public Health Program, which was initiated in 2009, the Chinese government makes an annual budget on a per capita basis to deliver a set of public health services to the rural population free of charge. These services include five routine antenatal care consultations to pregnant women in rural areas, including a list of basic services in accordance with the WHO’s focused ANC model. Although the program has a unified financing framework, its implementation strategies vary across provinces. For example, Shaanxi and Ningxia Provinces adopted voucher-based systems to deliver five routine antenatal consultations. Along with the consultations, a set of specific examinations and tests were included in the free service packages. In the program, community health providers distributed antenatal care vouchers with which the pregnant women could use to choose any local certificated hospital or health centre for five routine antenatal care visits, and receive the listed services free of charge. In each county, a county project office was responsible for keeping track of the vouchers to evaluate each health provider’s performance and allocate the government funds budgeted for the free services (Appendix table 1).

1. **The Extended Antenatal Care program**

**1) Supply-side efforts for integration**

Shaanxi Province piloted the Extended Antenatal Care Program to better integrate prenatal screen interventions into routine antenatal care. In the program, a series of supply-side efforts were made to establish mechanisms linking families, community health providers, and county-level facility-based services to deliver integrated antenatal care services. First, the provincial government introduced administrative and technical guidelines that guided and regulated the service procedures for the continuum of antenatal care. Community health providers were responsible for advocating and conducting health education and basic consultation, collecting pregnancy-related information, maintaining antenatal records, distributing antenatal vouchers, informing families of timely interventions, and facilitating referrals. County-level health providers were responsible for training, performing specialized consultations and B-ultrasound screening, and collecting blood samples and transporting them to the prefectural medical centre for foetal aneuploidies tests. The prefectural medical centres also received and managed referrals. Second, managerial functions were decentralized to the county level by setting up project offices located in the counties’ maternal and child health hospitals. This office coordinated related health providers and various sources of public funds. It set targets and held regular meetings with health providers to evaluate their performance and allocate funds. Third, a trainee-training approach was used. County experts were first trained by provincial experts on the guidelines and protocols and then sent to train local providers at the county and township level. Fourth, a unified information system was set up. Standard sheets were made for each specific health provider to monitor the service inputs, outputs, and outcomes. The county project offices assumed the responsibility to manage this and report to the provincial government.

**2) Demand-side subsidies to the extended interventions**

Shaanxi’s Extended Antenatal Care Program has several phases. Given fiscal limitations, in the pilot stage, which this study is evaluating, the provincial government selected one quarter (25 out of 107) of the rural counties to implement demand-side subsidies for the extended interventions, prioritising poor counties and those having maternal mortality above the provincial average. In the pilot, 190 RMB (about 22 US dollars) was budgeted for each woman to uptake the 2 extended prenatal screening services. These subsidies were distributed using the existing voucher-based system (Appendix Table 1).

**Appendix Table 2 Characteristics of the mothers that were investigated in six rural counties from Shaanxi and Ningxia Provinces, Western China, by regions, 2009-2016**

|  | **Shaanxi** | | | **Shaanxi** | | | **Ningxia** | | |
| --- | --- | --- | --- | --- | --- | --- | --- | --- | --- |
|  | **plain regions** | | | **mountainous regions** | | | **mountainous regions** | | |
|  | **N** | **%  unweighted** | **%  weighted** | **N** | **%  unweighted** | **%  weighted** | **N** | **%  unweighted** | **%  weighted** |
| **Date of delivery** | | | | | | | | | |
| Before May 1, 2015 | 222 | 45.1 | 79.8 | 239 | 45 | 78.4 | 254 | 44.3 | 78.2 |
| After May 1, 2015 | 270 | 54.9 | 20.2 | 292 | 55 | 21.6 | 320 | 55.8 | 21.8 |
| Missing | 0 | 0 |  | 0 | 0 |  | 0 | 0 |  |
| **Income tertile** | | | | | | | | | |
| Q1 | 92 | 18.7 | 20.2 | 125 | 23.5 | 24.5 | 314 | 54.7 | 51.8 |
| Q2 | 189 | 38.4 | 40.8 | 178 | 33.5 | 34.0 | 164 | 28.6 | 32.2 |
| Q3 | 208 | 42.3 | 38.9 | 227 | 42.8 | 41.6 | 96 | 16.7 | 16.1 |
| Missing | 3 | 0.6 |  | 1 | 0.2 |  | 0 | 0 |  |
| **Travel time to the county center** | | | | | | | | | |
| <=30 min | 264 | 53.7 | 51.6 | 57 | 10.7 | 10.8 | 194 | 33.8 | 33.9 |
| 30-90 min | 221 | 44.9 | 47.3 | 308 | 58.0 | 57.5 | 287 | 50 | 49.4 |
| >90 min | 7 | 1.4 | 1.2 | 166 | 31.3 | 31.7 | 93 | 16.2 | 16.7 |
| Missing | 0 | 0 |  | 0 | 0 |  | 0 | 0 |  |
| **Mother's education level** | | | | | | | | | |
| Illiterate | 3 | 0.6 | 1.1 | 30 | 5.7 | 6.4 | 60 | 10.5 | 11.8 |
| Primary | 266 | 54.1 | 58.1 | 363 | 68.4 | 69.8 | 458 | 79.8 | 80.5 |
| Secondary | 139 | 28.3 | 28.4 | 77 | 14.5 | 12.9 | 36 | 6.3 | 5.3 |
| College and above | 81 | 16.5 | 12.4 | 59 | 11.1 | 10.9 | 16 | 2.8 | 2.5 |
| Missing | 3 | 0.6 |  | 2 | 0.4 |  | 4 | 0.7 |  |
| **Mother's age at birth** | | | | | | | | | |
| <20 | 7 | 1.4 | 0.9 | 18 | 3.4 | 4.5 | 97 | 16.9 | 16.8 |
| 20- | 332 | 67.5 | 67.8 | 327 | 61.6 | 61.5 | 373 | 65 | 68.6 |
| 30- | 152 | 30.9 | 31.3 | 184 | 34.7 | 34.0 | 99 | 17.3 | 14.6 |
| Missing | 1 | 0.2 |  | 2 | 0.4 |  | 5 | 0.9 |  |
| **Parity at birth** | | | | | | | | | |
| 1 | 271 | 55.1 | 52.0 | 207 | 39 | 39.0 | 171 | 29.8 | 29.4 |
| 2 | 215 | 43.7 | 46.8 | 299 | 56.3 | 57.2 | 239 | 41.6 | 42.2 |
| 3 and above | 5 | 1 | 1.1 | 24 | 4.5 | 3.8 | 162 | 28.2 | 28.5 |
| Missing | 1 | 0.2 |  | 1 | 0.2 |  | 2 | 0.4 |  |
| **Place of delivery (in home county)** | | | | | | | | | |
| No | 47 | 9.6 | 8.0 | 47 | 8.9 | 11.0 | 40 | 7 | 5.4 |
| Yes | 444 | 90.2 | 92.0 | 482 | 90.8 | 89.1 | 533 | 92.9 | 94.6 |
| Missing | 1 | 0.2 |  | 2 | 0.4 |  | 1 | 0.2 |  |
| **Mother's NCMS coverage** | | | | | | | | | |
| No | 27 | 5.5 | 4.8 | 29 | 5.5 | 5.5 | 46 | 8 | 7.9 |
| Yes | 462 | 93.9 | 95.2 | 500 | 94.2 | 94.5 | 526 | 91.6 | 92.1 |
| Missing | 3 | 0.6 |  | 2 | 0.4 |  | 2 | 0.4 |  |
| **County** | | | | | | | | | |
| Mei | 244 | 49.6 | 50.2 |  |  |  |  |  |  |
| Qishan | 248 | 50.4 | 49.8 |  |  |  |  |  |  |
| Baihe |  |  |  | 259 | 48.8 | 48.5 |  |  |  |
| Xunyang |  |  |  | 272 | 51.2 | 51.5 |  |  |  |
| Tongxin |  |  |  |  |  |  | 271 | 47.2 | 47.6 |
| Yuanzhou |  |  |  |  |  |  | 303 | 52.8 | 52.4 |
| Missing |  |  |  |  |  |  |  |  |  |

**Appendix Table 3 Factors affecting the coverage of routine antenatal care and the extended prenatal screening interventions among the mothers that were investigated in four rural counties from Shaanxi Province, Western China, 2009-2016**

|  |  | **Prenatal fetal** | | | **Prenatal B** | | | **At least five routine** | | |
| --- | --- | --- | --- | --- | --- | --- | --- | --- | --- | --- |
|  |  | **aneuploidies screening** | | | **ultrasound screening** | | | **antenatal visits** | | |
|  | **N** | % | Crude RR | Adjust RR | % | Crude RR | Adjust RR | % | Crude RR | Adjust RR |
|  |  |  | (95% CI) | (95% CI) |  | (95% CI) | (95% CI) |  | (95% CI) | (95% CI) |
| **Date of delivery** | | | | | | | | | | |
| Before May 1, 2015 | 461 | 44% | 1 | 1 | 52% | 1 | 1 | 82% | 1 | 1 |
| After May 1, 2015 | 562 | 70% | 1.56 | 1.52 | 76% | 1.45 | 1.44 | 93% | 1.14 | 1.12 |
|  |  |  | 1.41, 1.77 | 1.36, 1.70 |  | 1.27, 1.67 | 1.31, 1.58 |  | 0.98, 1.32 | 0.97, 1.29 |
| **Mother's education level** | | | | | | | | | | |
| Primary and below | 662 | 45% | 1 | 1 | 55% | 1 | 1 | 80% | 1 | 1 |
| Secondary and above | 356 | 60% | 1.33 | 1.20 | 63% | 1.18 | 1.12 | 94% | 1.17 | 1.10 |
|  |  |  | 1.22, 1.45 | 1.09, 1.33 |  | 1.09, 1.29 | 1.02, 1.23 |  | 1.07, 1.28 | 1.03, 1.18 |
| **Income quintile** | | | | | | | | | | |
| Q1 | 340 | 45% | 1 | 1 | 56% | 1 | 1 | 79% | 1 | 1 |
| Q2 | 346 | 51% | 1.13 | 0.98 | 54% | 0.97 | 0.96 | 85% | 1.07 | 1.03 |
|  |  |  | 0.73, 1.74 | 0.87, 1.11 |  | 0.71, 1.35 | 0.87, 1.07 |  | 0.92, 1.26 | 0.90, 1.19 |
| Q3 | 333 | 54% | 1.20 | 1.02 | 62% | 1.12 | 1 | 88% | 1.11 | 1.06 |
|  |  |  | 0.95, 1.52 | 0.91, 1.16 |  | 0.80, 1.57 | 0.90, 1.11 |  | 0.85, 1.46 | 0.83, 1.35 |
| **Mother's NCMS coverage** | | | | | | | | | | |
| No | 56 | 47% | 1 | 1 | 50% | 1 | 1 | 78% | 1 | 1 |
| Yes | 962 | 50% | 1.07 | 1.15 | 58% | 1.15 | 1.08 | 85% | 1.09 | 1.12 |
|  |  |  | 0.47, 2.43 | 0.90, 1.47 |  | 0.79, 1.69 | 0.90, 1.29 |  | 0.66, 1.81 | 0.68, 1.84 |
| **Travel time to the county center** | | | | | | | | | | |
| <=30 min | 321 | 56% | 1 | 1 | 61% | 1 | 1 | 88% | 1 | 1 |
| 30-90 min | 529 | 48% | 0.86 | 0.93 | 56% | 0.93 | 0.93 | 86% | 0.97 | 1.02 |
|  |  |  | 0.69, 1.07 | 0.83, 1.03 |  | 0.74, 1.16 | 0.84, 1.03 |  | 0.84, 1.13 | 0.93, 1.11 |
| >=90 min | 173 | 43% | 0.77 | 0.83 | 53% | 0.87 | 0.91 | 73% | 0.82 | 0.93 |
|  |  |  | 0.55, 1.08 | 0.67, 1.03 |  | 0.74, 1.03 | 0.78, 1.07 |  | 0.68, 1.00 | 0.84, 1.03 |
| **Parity at birth** | | | | | | | | | | |
| 1 | 478 | 56% | 1 | 1 | 61% | 1 | 1 | 90% | 1 | 1 |
| 2 and above | 543 | 45% | 0.81 | 0.88 | 54% | 0.89 | 0.86 | 80% | 0.89 | 0.94 |
|  |  |  | 0.68, 0.97 | 0.79, 0.99 |  | 0.70, 1.12 | 0.78, 0.95 |  | 0.79, 1.01 | 0.82, 1.08 |
| **Place of delivery (in home county)** | | | | | | | | | | |
| No | 94 | 60% | 1 | 1 | 63% | 1 | 1 | 85% | 1 | 1 |
| Yes | 926 | 49% | 0.81 | 0.77 | 57% | 0.89 | 0.89 | 84% | 0.98 | 0.95 |
|  |  |  | 0.67, 0.98 | 0.68, 0.87 |  | 0.88, 0.91 | 0.78, 1.01 |  | 0.83, 1.16 | 0.80, 1.12 |
| **Mother's age at birth** | | | | | | | | | | |
| <30 | 684 | 54% | 1 | 1 | 59% | 1 | 1 | 86% | 1 | 1 |
| 30- | 336 | 42% | 0.77 | 0.9 | 55% | 0.94 | 0.99 | 80% | 0.93 | 0.99 |
|  |  |  | 0.61, 0.99 | 0.79, 1.02 |  | 0.84, 1.05 | 0.89, 1.10 |  | 0.90, 0.97 | 0.91, 1.06 |
| **County** | | | | | | | | | | |
| Mei | 244 | 61% | 1 | 1 | 51% | 1 | 1 | 95% | 1 | 1 |
|  |  |  | 0.75 | 0.83 |  | 1.04 | 1.08 |  | 0.90 | 0.90 |
| Qishan | 248 | 45% | 0.74, 0.75 | 0.73, 0.94 | 53% | 1.04, 1.04 | 0.95, 1.24 | 86% | 0.90, 0.90 | 0.88, 0.93 |
|  |  |  | 0.81 | 0.97 |  | 1.36 | 1.42 |  | 0.86 | 0.88 |
| Baihe | 259 | 49% | 0.80, 0.81 | 0.85, 1.11 | 69% | 1.36, 1.36 | 1.24, 1.61 | 82% | 0.86, 0.86 | 0.84, 0.92 |
|  |  |  | 0.73 | 0.79 |  | 1.06 | 1.17 |  | 0.79 | 0.83 |
| Xunyang | 272 | 44% | 0.72, 0.73 | 0.66, 0.95 | 54% | 1.06, 1.06 | 1.00, 1.37 | 75% | 0.79, 0.79 | 0.80, 0.87 |

**Notes：**

1. The included number of mothers that gave birth during the 2009-2016 period in the four rural counties from Shaanxi province was 1023. The sampling design was adjusted using the “svy” command in STATA.
2. Since May 1, 2015, Shaanxi province has been pushing the Extended Antenatal Care Program that integrates congenital abnormality screening interventions into routine antenatal care.
3. Poisson regression was used to analyze the factors affecting coverage. Sandwich robust standard errors were estimated to calculate 95% confidence intervals (CIs). The adjusted covariates include date of delivery, mothers’ educational achievement, households’ income per capita tertiles, mothers' NCMS coverage at birth, travel time to the county center, mothers’ parity at birth, whether the women delivered in their home county, mothers’ age at birth, and county dummy variables.
4. *Prenatal fetal aneuploidies screening*, *prenatal B-ultrasound screening,* and *at least five routine antenatal visits* were measurements of actual coverage for these interventions.

**Appendix Table 4 A difference-in-difference estimation of the impact of the supply-side policy of Shaanxi’s Extended Antenatal Care Program on awareness, benefit, utilization, and costs of the antenatal care services, with Ningxia province as the control group, by mothers’ educational achievement, using data from 4 rural counties in Shaanxi and Ningxia Provinces, Western China, 2009-2016**

|  | **Shaanxi** | | | **Ningxia** | | | **Difference-in-difference** | |
| --- | --- | --- | --- | --- | --- | --- | --- | --- |
|  | Before  the Program | After  the Program | Diff. | Before  the Program | After  the Program | Diff. | Rate/RMB | P value |
| **Overall** |  |  |  |  |  |  |  |  |
| **Voucher-related performance** |  |  |  |  |  |  |  |  |
| Knowing about the five free antenatal care visits | 78.0% | 85.9% | 7.9% | 76.4% | 78.1% | 1.7% | 2.6 | 0.5887 |
| Having used antenatal care vouchers | 78.8% | 88.0% | 9.2% | 79.1% | 83.1% | 4.0% | 2.7 | 0.5591 |
| **Service-related performance** |  |  |  |  |  |  |  |  |
| At least five routine antenatal visits | 78.0% | 89.8% | 11.8% | 66.1% | 78.8% | 12.6% | -4.0 | 0.4079 |
| Prenatal fetal aneuploidies screening | 40.3% | 64.8% | 24.5% | 10.2% | 14.7% | 4.5% | 18.5 | 0.0003 |
| Prenatal B-ultrasound screening | 47.5% | 78.9% | 31.4% | 48.8% | 49.1% | 0.2% | 32.6 | 0.0000 |
| **Cost-related performance** |  |  |  |  |  |  |  |  |
| ANC medical cost (median) | 610.5 | 1500.0 | 889.5 | 156.3 | 104.2 | -52.1 | 753.2 | 0.0000 |
| ANC non-medical cost (median) | 318.2 | 500.0 | 181.9 | 200.0 | 100.6 | -99.4 | 153.6 | 0.0000 |
| **Mother's education - primary and below** | |  |  |  |  |  |  |  |
| **Voucher-related performance** |  |  |  |  |  |  |  |  |
| Knowing about the five free antenatal care visits | 73.9% | 85.5% | 11.6% | 76.6% | 79.2% | 2.6% | 4.8 | 0.2279 |
| Having used antenatal care vouchers | 75.8% | 88.1% | 12.3% | 79.6% | 84.8% | 5.2% | -0.3 | 0.9341 |
| **Service-related performance** |  |  |  |  |  |  |  |  |
| At least five routine antenatal visits | 73.2% | 84.9% | 11.7% | 64.3% | 80.2% | 16.0% | 3.4 | 0.3985 |
| Prenatal fetal aneuploidies screening | 39.5% | 49.7% | 10.2% | 8.9% | 13.4% | 4.5% | 35.0 | 0.0000 |
| Prenatal B-ultrasound screening | 45.9% | 71.1% | 25.2% | 47.7% | 47.3% | -0.3% | 27.1 | 0.0000 |
| **Cost-related performance** |  |  |  |  |  |  |  |  |
| ANC medical cost (median) | 539.5 | 1012.0 | 472.5 | 156.3 | 197.3 | 41.0 | 847.0 | 0.0000 |
| ANC non-medical cost (median) | 312.6 | 500.0 | 187.4 | 104.2 | 100.0 | -4.2 | 255.8 | 0.0000 |
| **Mother's education - secondary and above** | |  |  |  |  |  |  |  |
| **Voucher-related performance** |  |  |  |  |  |  |  |  |
| Knowing about the five free antenatal care visits | 89.5% | 86.4% | -3.1% | 72.2% | 67.6% | -4.6% | 15.2 | 0.0672 |
| Having used antenatal care vouchers | 88.2% | 88.0% | -0.2% | 72.2% | 67.6% | -4.6% | 17.2 | 0.0405 |
| **Service-related performance** |  |  |  |  |  |  |  |  |
| At least five routine antenatal visits | 89.5% | 96.0% | 6.5% | 88.9% | 70.6% | -18.3% | 20.2 | 0.0027 |
| Prenatal fetal aneuploidies screening | 43.4% | 84.0% | 40.6% | 27.8% | 26.5% | -1.3% | 58.0 | 0.0000 |
| Prenatal B-ultrasound screening | 51.3% | 88.8% | 37.5% | 66.7% | 58.8% | -7.8% | 29.6 | 0.0016 |
| **Cost-related performance** |  |  |  |  |  |  |  |  |
| ANC medical cost (median) | 773.9 | 2000.0 | 1226.1 | 210.2 | 500.0 | 289.8 | 1373.2 | 0.0000 |
| ANC non-medical cost (median) | 343.0 | 500.0 | 157.0 | 109.6 | 280.0 | 170.5 | 220.6 | 0.0449 |

**Notes:**

1. The number of included mothers that gave birth during the 2009-2016 period in four rural counties from Shaanxi and Ningxia provinces, Western China, was 1094. The sampling design was adjusted using the “svy” command in STATA. In the Extended Maternal Health Care Program, the government of Shaanxi adopted both supply- and demand-side policies. Since May 2015, supply-side measures have been universally conducted in Shaanxi, while demand-side measures were adopted in selected counties. In order to estimate the impact of the supply-side policies of the Program, we included two counties – Qishan and Xunyang - from Shaanxi where only supply-side measures were conducted in the treatment group and two counties from Ningxia, Shaanxi’s neighboring province, in the control group in this analysis.
2. The difference-in-difference estimation was fulfilled using a regression analysis that incorporated a set of dummy variables, i.e., variables indicating treatment, timing of the treatment, and their interaction term. The policy’s time of initiation was set as May 1, 2015. The coefficient of this interaction term is a difference-in-difference estimation of the policy’s impact. Additionally, we adjusted the following covariates: mothers’ educational achievement, households’ income per capita tertiles, mothers' NCMS coverage at birth, travel time to the county center, mothers’ parity at birth, whether the women delivered in their home county, and mothers’ age at birth. We used ordinary least squares to model awareness and coverage; we used quantile regression to model costs in order to estimate the median.
3. *Knowing about the five free antenatal care visits* measured the proportion of mothers who were aware of the five routine antenatal care services provided by the government free. *Having used antenatal care vouchers* was calculated as the proportion of mothers who declared having used the antenatal care vouchers. *At least five antenatal visits*, *prenatal fetal aneuploidies screening*, and *prenatal B-ultrasound screening* were measurements of the actual coverage for these interventions. *ANC medical cost* was the median of the out-of-pocket payments that women declared were made to the health providers while using the antenatal care services. In addition, *ANC non-medical cost* was the median of the costs incurred by the women for their travel and accommodations while using the antenatal care services.

**Appendix Table 5 A difference-in-difference estimation of the impact of the demand-side policy of Shaanxi’s Extended Antenatal Care Program on awareness, benefit, utilization, and costs of the antenatal care services, with a neighboring county within Shaanxi as the control group, using data from 2 rural counties in Shaanxi Province’s mountainous areas, Western China, 2009-2016**

|  | **Baihe** | | | | **Xunyang** | | | **Difference-in-difference** | | |
| --- | --- | --- | --- | --- | --- | --- | --- | --- | --- | --- |
|  | Before  the Program | After  the Program | Diff. | Before  the Program | | After  the Program | Diff. | | Rate/RMB | P value |
| **Voucher-related performance** |  |  |  |  | |  |  | |  |  |
| Knowing about the five free antenatal care visits | 44.7% | 75.9% | 31.1% | 79.2% | | 84.4% | 5.2% | | 24.4 | 0.0011 |
| Having used antenatal care vouchers | 58.8% | 85.5% | 26.7% | 78.4% | | 88.4% | 10.0% | | 16.5 | 0.0172 |
| Knowing about the Extended Antenatal Care Program | 29.1% | 78.2% | 49.1% | 34.1% | | 32.0% | -2.2% | | 50.8 | 0.0000 |
| Having used the extended interventions free of charge | 15.8% | 32.4% | 16.6% | 25.6% | | 17.0% | -8.6% | | 24.9 | 0.0007 |
| **Service-related performance** |  |  |  |  | |  |  | |  |  |
| At least five routine antenatal visits | 78.1% | 94.5% | 16.4% | 74.4% | | 81.0% | 6.6% | | 9.1 | 0.1751 |
| Prenatal fetal aneuploidies screening | 41.2% | 78.6% | 37.4% | 42.4% | | 52.4% | 10.0% | | 25.5 | 0.0024 |
| Prenatal B-ultrasound screening | 66.7% | 86.9% | 20.2% | 48.8% | | 76.2% | 27.4% | | -9.4 | 0.2257 |
| **Cost-related performance** |  |  |  |  | |  |  | |  |  |
| ANC medical cost (median) | 1095.5 | 2000.0 | 904.5 | 607.2 | | 1012.0 | 404.8 | | 204.2 | 0.4344 |
| ANC non-medical cost (median) | 309.8 | 300.0 | -9.8 | 431.6 | | 500.0 | 68.4 | | -99.2 | 0.1569 |

**Notes:**

1. In this analysis, 531 mothers that gave birth during the 2009-2016 period in Baihe and Xunyang, two neighboring counties from the mountainous areas of Shaanxi, were included. In Baihe, both supply- and demand- side measures were conducted in the Extended Antenatal Care Program, while in Xunyang, only supply-side measures were conducted. The sampling design was adjusted using the “svy” command in STATA.
2. The analytical approach is the same as that of Table 3 in terms of model specification, regression methods, and adjusted covariates.
3. The definition of dependent variables is the same as that in Table 3. Additionally, *knowing about the Extended Antenatal Care Program* measured the proportion of mothers who were aware of the Program. *Having used the extended interventions free of charge* measured the proportion of mothers who declared benefits.

**Appendix Table 6 A difference-in-difference estimation of the impact of the demand-side policy of Shaanxi’s Extended Antenatal Care Program on awareness, benefit, utilization, and costs of the antenatal care services, with a neighboring county within Shaanxi as the control group, using data from 2 rural counties in Shaanxi Province’s plain areas, Western China, 2009-2016**

|  | **Mei** | | | **Qishan** | | | **Difference-in-difference** | |
| --- | --- | --- | --- | --- | --- | --- | --- | --- |
|  | Before  intervention | After  intervention | Diff. | Before  intervention | After  intervention | Diff. | Rate/RMB | P value |
| **Voucher-related performance** |  |  |  |  |  |  |  |  |
| Knowing about the five free antenatal care visits | 90.3% | 90.1% | -0.2% | 76.6% | 87.6% | 11.0% | -10.9 | 0.0710 |
| Having used antenatal care vouchers | 93.8% | 90.8% | -3.0% | 79.3% | 87.6% | 8.3% | -10.3 | 0.0657 |
| Knowing about the Extended Antenatal Care Program | 39.3% | 55.8% | 16.5% | 29.9% | 45.6% | 15.7% | 1.4 | 0.8777 |
| Having used the extended interventions free of charge | 31.9% | 39.7% | 7.8% | 20.7% | 23.4% | 2.6% | 5.5 | 0.5067 |
| **Service-related performance** |  |  |  |  |  |  |  |  |
| At least five routine antenatal visits | 94.7% | 94.7% | 0.0% | 82.0% | 99.3% | 17.3% | -15.0 | 0.0010 |
| Prenatal fetal aneuploidies screening | 60.2% | 76.3% | 16.2% | 37.8% | 78.1% | 40.3% | -19.8 | 0.0182 |
| Prenatal B-ultrasound screening | 48.7% | 67.2% | 18.5% | 45.9% | 81.8% | 35.8% | -14.1 | 0.0998 |
| **Cost-related performance** |  |  |  |  |  |  |  |  |
| ANC medical cost (median) | 1023.0 | 1500.0 | 477.0 | 613.8 | 2000.0 | 1386.2 | -511.5 | 0.0000 |
| ANC non-medical cost (median) | 235.0 | 400.0 | 165.0 | 182.2 | 360.0 | 177.8 | 27.0 | 0.7243 |

**Notes:**

1. In this analysis, 492 mothers that gave birth during the 2009-2016 period in Mei and Qishan, two neighboring counties from the plain areas of Shaanxi, were included. In Mei, both supply- and demand-side measures were conducted in the Extended Antenatal Care Program, while in Qishan, only supply-side measures were conducted. The sampling design was adjusted using the “svy” command in STATA.
2. The analytical approach used and the definitions of the variables are the same as those in Table 4.

**Appendix Table 7 A difference-in-difference estimation of the impact of Shaanxi’s Extended Antenatal Care Program demand-side policy on awareness, benefit, utilization, and costs of the antenatal care services, by region and mother’s educational achievement, using data from four rural counties in Shaanxi Province, Western China, 2009-2016**

|  | **Mountainous regions of Shaanxi** | | | | **Plain regions of Shaanxi** | | | |
| --- | --- | --- | --- | --- | --- | --- | --- | --- |
|  | **Mother's education  - primary and below (N=390)** | | **Mother's education  - secondary and above (N=134)** | | **Mother's education  - primary and below (N=269)** | | **Mother's education  - secondary and above (N=220)** | |
|  | Rate/RMB | P value | Rate/RMB | P value | Rate/RMB | P value | Rate/RMB | P value |
| **Voucher-related performance** |  |  |  |  |  |  |  |  |
| Knowing about the five free antenatal care visits | 21.4 | 0.0180 | 26.0 | 0.0582 | -13.3 | 0.0985 | -7.2 | 0.4464 |
| Having used antenatal care vouchers | 17.1 | 0.0365 | 12.0 | 0.3848 | -12.9 | 0.0938 | -7.3 | 0.3718 |
| Knowing about the Extended Antenatal Care Program | 48.3 | 0.0000 | 49.9 | 0.0057 | 3.3 | 0.7834 | 2.5 | 0.8652 |
| Having used the extended interventions free of charge | 24.2 | 0.0032 | 16.6 | 0.3575 | 13.0 | 0.2352 | -1.9 | 0.8905 |
| **Service-related performance** |  |  |  |  |  |  |  |  |
| At least five antenatal visits | 6.5 | 0.4312 | 13.0 | 0.2718 | -17.6 | 0.0099 | -12.7 | 0.0101 |
| Prenatal fetal aneuploidies screening | 34.3 | 0.0005 | 0.0 | 1.0000 | -8.4 | 0.4807 | -27.1 | 0.0289 |
| Prenatal B-ultrasound screening | -5.0 | 0.5872 | -21.8 | 0.1405 | -12.8 | 0.2903 | -14.1 | 0.2685 |
| **Cost-related performance** |  |  |  |  |  |  |  |  |
| ANC medical cost (median) | 318.2 | 0.2495 | -51.4 | 0.9452 | -424.0 | 0.0420 | -572.8 | 0.0073 |
| ANC non-medical cost (median) | -36.3 | 0.5804 | -227.7 | 0.3193 | -10.1 | 0.9106 | 16.8 | 0.8863 |

**Notes:**

1. In this analysis, 1023 mothers that gave birth during the 2009-2016 period in four counties from Shaanxi Province, Western China were included. The sampling design was adjusted using the “svy” command in STATA.
2. The analytical approach used and the definitions of the variables are the same as those in Table 4.

**Appendix Figure 1 Location of the Shaanxi and Ningxia Provinces, the 6 counties and the townships that were investigated**


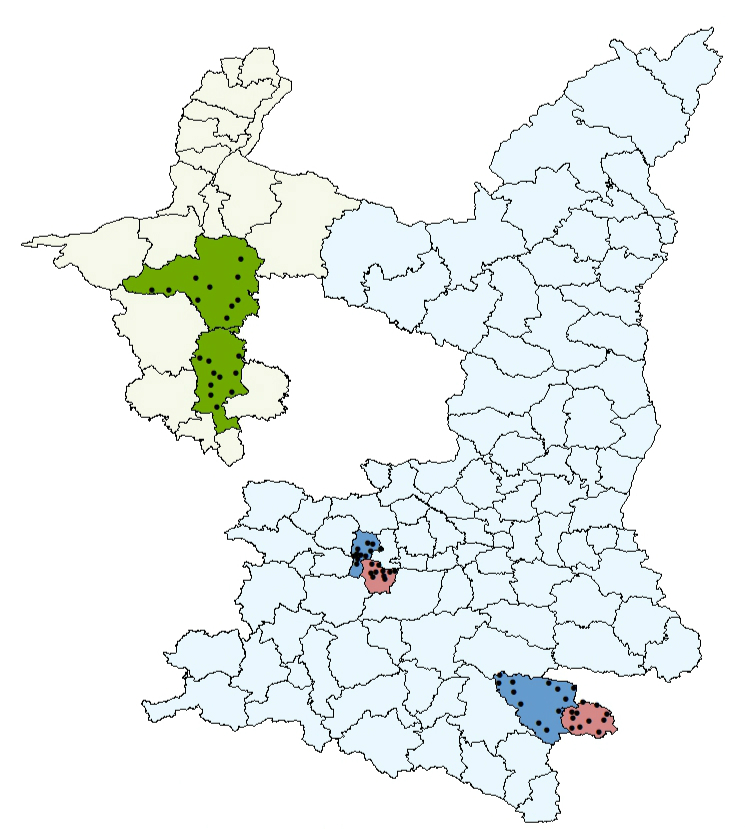

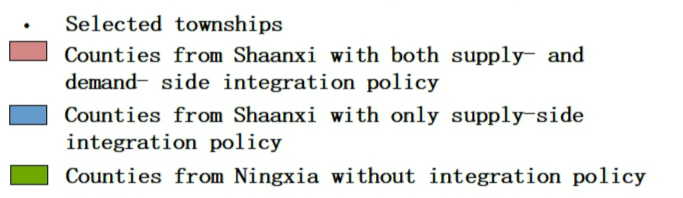

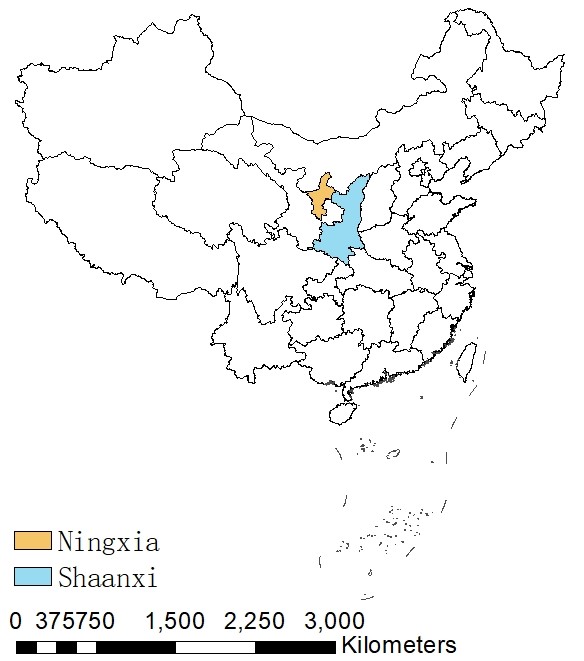


**Plain regions**

**Mountainous regions**

**Appendix Figure 2 Pre- and post-program time series on awareness, benefit, utilization, and costs of the antenatal care services**

1. **Impact of the supply-side integration policy**
2. **Overall**
3. **Knowing 5 free antenatal care visits**

**2) Having used antenatal care vouchers**

**3) Prenatal foetal aneuploidies screening**

**4) Prenatal B ultrasound screening**

**5) At least five routine antenatal visits**

**6) ANC medical costs (median)**

**7) ANC non-medical costs (median)**

1. **Amongst women with primary and below education**

**1) Knowing 5 free antenatal care visits**

**2) Having used antenatal care vouchers**

**3) Prenatal foetal aneuploidies screening**

**4) Prenatal B ultrasound screening**

**5) At least five routine antenatal visits**

**6) ANC medical costs (median)**

**7) ANC non-medical costs (median)**

1. **Amongst women with secondary and above education**

**1) Knowing 5 free antenatal care visits**

**2) Having used antenatal care vouchers**

**3) Prenatal foetal aneuploidies screening**

**4) Prenatal B ultrasound screening**

**5) At least five routine antenatal visits**

**6) ANC medical costs (median)**

**7) ANC non-medical costs (median)**

1. **Impact of the demand-side integration policy**

**Context: Mountainous regions of Shaanxi**

1. **Overall**

**1) Knowing 5 free antenatal care visits**

**2) Having used antenatal care vouchers**

**3) Knowing the Extended Antenatal Health Care Program**

**4) Having used the extended interventions free of charge**

**5) Prenatal foetal aneuploidies screening**

**6) Prenatal B ultrasound screening**

**7) At least five routine antenatal visits**

**8) ANC medical costs (median)**

**9) ANC non-medical costs (median)**

1. **Amongst women with primary and below education**

**1) Knowing 5 free antenatal care visits**

**2) Having used antenatal care vouchers**

**3) Knowing the Extended Antenatal Health Care Program**

**4) Having used the extended interventions free of charge**

**5) Prenatal foetal aneuploidies screening**

**6) Prenatal B ultrasound screening**

**7) At least five routine antenatal visits**

**8) ANC medical costs (median)**

**9) ANC non-medical costs (median)**

1. **Amongst women with secondary and above education**

**1) Knowing 5 free antenatal care visits**

**2) Having used antenatal care vouchers**

**3) Knowing the Extended Antenatal Health Care Program**

**4) Having used the extended interventions free of charge**

**5) Prenatal foetal aneuploidies screening**

**6) Prenatal B ultrasound screening**

**7) At least five routine antenatal visits**

**8) ANC medical costs (median)**

**9) ANC non-medical costs (median)**

1. **Impact of the demand-side integration policy**

**Context: Plain regions of Shaanxi**

1. **Overall**

**1) Knowing 5 free antenatal care visits**

**2) Having used antenatal care vouchers**

**3) Knowing the Extended Antenatal Health Care Program**

**4) Having used the extended interventions free of charge**

**5) Prenatal foetal aneuploidies screening**

**6) Prenatal B ultrasound screening**

**7) At least five routine antenatal visits**

**8) ANC medical costs (median)**

**9) ANC non-medical costs (median)**

1. **Amongst women with primary and below education**

**1) Knowing 5 free antenatal care visits**

**2) Having used antenatal care vouchers**

**3) Knowing the Extended Antenatal Health Care Program**

**4) Having used the extended interventions free of charge**

**5) Prenatal foetal aneuploidies screening**

**6) Prenatal B ultrasound screening**

**7) At least five routine antenatal visits**

**8) ANC medical costs (median)**

**9) ANC non-medical costs (median)**

1. **Amongst women with secondary and above education**

**1) Knowing 5 free antenatal care visits**

**2) Having used antenatal care vouchers**

**3) Knowing the Extended Antenatal Health Care Program**

**4) Having used the extended interventions free of charge**

**5) Prenatal foetal aneuploidies screening**

**6) Prenatal B ultrasound screening**

**7) At least five routine antenatal visits**

**8) ANC medical costs (median)**

**9) ANC non-medical costs (median)**
