# Supplementary material for: Evaluation of a pilot program that integrated prenatal screening into routine antenatal care in western rural China: an interrupted time-series study
Source: Lancet Reg Health West Pac. 2020 Dec 24;6:100075. doi: 10.1016/j.lanwpc.2020.100075 (PMC8315490; doi:10.1016/j.lanwpc.2020.100075)
Supplement: Supplementary file 3 [file mmc3.docx]

**摘要**

**背景**

本研究评价了中国西部农村地区开展的一项大规模试点项目，该项目将出生缺陷筛查服务整合入国家基本公共卫生项目的常规产前检查。

**方法**

研究调查了2009-2016年期间有分娩史的1597名妇女。采用分断时间序列设计，通过对比陕西省2个开展了供给面改革的县和相邻的宁夏自治区没有相应政策的2个县，以及陕西省内既开展供给面改革又进行需求面补贴的2个县和该省2个仅开展了供给面改革的县，评价了该试点项目对政府项目的知晓、产前筛查服务的覆盖和产检费用的影响。分析中调整了抽样和妇女的个体特征，并按妇女的文化程度进行了亚组分析。

**发现**

项目执行一年以后，产前血清学和超声筛查的覆盖率分别升高了25.0和23.5个百分点。项目供给面的政策提高两项筛查服务的覆盖率达17.2 (90% CI 7.84-26.65%)和27.3个百分点(90%CI 16.15-38.51%)，并使得产前检查总费用中位数增加796.5元(90% CI 595.50-997.52)。这些政策效果在高中及以上文化程度组妇女中更为显著。但是，该项目的需求面补贴政策，即公共卫生券，似乎只在陕西省的山区更有效果。在大巴山区，该项目提高了产妇对相关项目的知晓和受益，使得血清学筛查的覆盖率提高了28.6个百分点(90% CI 13.40-43.79%)，且没有增加产前检查总费用。这些政策在初中及以下文化程度组妇女中更为显著。另外，在仅执行供给面改革的地区，服务的教育相关不公平性在项目执行后有所扩大；但在同时推行了需求面补贴的地区，服务覆盖的教育差异却消失了。

**解释**

陕西省2015年开展的孕产妇系统保健免费基本服务项目为中国其他农村地区推进整合型的妇幼卫生项目提供了有益借鉴。今后利用财政资金进行需求面补贴时可以专注于特定的地区和初中及以下的妇女进行定向扶持，以提高健康扶贫的效果。

**项目资助情况**

该研究得到了国家自然科学基金的资助(项目号71422009, 71761130083)

*Disclaimer: This translation in Chinese was submitted by the authors and we reproduce it as supplied. It has not been peer reviewed. Our editorial processes have only been applied to the original abstract in English, which should serve as reference for this manuscript.*
